# Effects of Cannabidiol on Exercise Physiology and Bioenergetics: A Randomised Controlled Pilot Trial

**DOI:** 10.1186/s40798-022-00417-y

**Published:** 2022-03-02

**Authors:** Ayshe Sahinovic, Christopher Irwin, Peter T. Doohan, Richard C. Kevin, Amanda J. Cox, Namson S. Lau, Ben Desbrow, Nathan A. Johnson, Angelo Sabag, Matthew Hislop, Paul S. Haber, Iain S. McGregor, Danielle McCartney

**Affiliations:** 1 Lambert Initiative for Cannabinoid Therapeutics, The University of Sydney, Sydney, NSW Australia; 2 Brain and Mind Centre, The University of Sydney, Sydney, NSW Australia; 3 School of Psychology, Faculty of Science, The University of Sydney, Sydney, NSW Australia; 4 School of Health Sciences and Social Work, Griffith University, Gold Coast, QLD Australia; 5 Menzies Health Institute Queensland, Gold Coast, QLD Australia; 6 School of Medical Science, Griffith University, Gold Coast, QLD Australia; 7 South West Clinical School, University of New South Wales, Sydney, NSW Australia; 8 Ingham Institute of Applied Medical Research, Sydney, NSW Australia; 9 Faculty of Medicine and Health, The University of Sydney, Sydney, NSW Australia; 10 NICM Health Research Institute, Western Sydney University, Sydney, NSW Australia; 11 Brisbane Sport and Exercise Medicine Specialists, Brisbane, QLD Australia; 12 Royal Prince Alfred Hospital, Sydney, NSW Australia

**Keywords:** Cannabis, Cannabinoid, Cannabidiol, CBD, Sport, Supplement

## Abstract

**Background:**

Cannabidiol (CBD) has demonstrated anti-inflammatory, analgesic, anxiolytic and neuroprotective effects that have the potential to benefit athletes. This pilot study investigated the effects of acute, oral CBD treatment on physiological and psychological responses to aerobic exercise to determine its practical utility within the sporting context.

**Methods:**

On two occasions, nine endurance-trained males (mean ± SD V̇O_2max_: 57.4 ± 4.0 mL·min^−1^·kg^−1^) ran for 60 min at a fixed intensity (70% V̇O_2max_) (RUN 1) before completing an incremental run to exhaustion (RUN 2). Participants received CBD (300 mg; oral) or placebo 1.5 h before exercise in a randomised, double-blind design. Respiratory gases (V̇O_2_), respiratory exchange ratio (RER), heart rate (HR), blood glucose (BG) and lactate (BL) concentrations, and ratings of perceived exertion (RPE) and pleasure–displeasure were measured at three timepoints (T1–3) during RUN 1. V̇O_2max_, RER_max_, HR_max_ and time to exhaustion (TTE) were recorded during RUN 2. Venous blood was drawn at Baseline, Pre- and Post-RUN 1, Post-RUN 2 and 1 h Post-RUN 2. Data were synthesised using Cohen’s *d*_z_ effect sizes and 85% confidence intervals (CIs). Effects were considered worthy of further investigation if the 85% CI included ± 0.5 but not zero.

**Results:**

CBD appeared to increase V̇O_2_ (T2: + 38 ± 48 mL·min^−1^, *d*_z_: 0.25–1.35), ratings of pleasure (T1: + 0.7 ± 0.9, *d*_z_: 0.22–1.32; T2: + 0.8 ± 1.1, *d*_z_: 0.17–1.25) and BL (T2: + 3.3 ± 6.4 mmol·L^−1^, *d*_z_: > 0.00–1.03) during RUN 1 compared to placebo. No differences in HR, RPE, BG or RER were observed between treatments. CBD appeared to increase V̇O_2max_ (+ 119 ± 206 mL·min^−1^, *d*_z_: 0.06–1.10) and RER_max_ (+ 0.04 ± 0.05 *d*_z_: 0.24–1.34) during RUN 2 compared to placebo. No differences in TTE or HR_max_ were observed between treatments. Exercise increased serum interleukin (IL)-6, IL-1β, tumour necrosis factor-α, lipopolysaccharide and myoglobin concentrations (i.e. Baseline vs. Post-RUN 1, Post-RUN 2 and/or 1-h Post-RUN 2, *p*’s < 0.05). However, the changes were small, making it difficult to reliably evaluate the effect of CBD, where an effect appeared to be present. Plasma concentrations of the endogenous cannabinoid, anandamide (AEA), increased Post-RUN 1 and Post-RUN 2, relative to Baseline and Pre-RUN 1 (*p*’s < 0.05). CBD appeared to reduce AEA concentrations Post-RUN 2, compared to placebo (− 0.95 ± 0.64 pmol·mL^−1^, *d*_z_: − 2.19, − 0.79).

**Conclusion:**

CBD appears to alter some key physiological and psychological responses to aerobic exercise without impairing performance. Larger studies are required to confirm and better understand these preliminary findings.

*Trial Registration* This investigation was approved by the Sydney Local Health District’s Human Research Ethics Committee (2020/ETH00226) and registered with the Australia and New Zealand Clinical Trials Registry (ACTRN12620000941965).

**Supplementary Information:**

The online version contains supplementary material available at 10.1186/s40798-022-00417-y.

## Key Points

- CBD (300 mg; oral) appears to alter physiological and psychological responses to aerobic exercise.
- The effects of CBD on submaximal (V̇O_2_) and maximal (V̇O_2max_) oxygen consumption, feelings of pleasure during exercise, and exercise-induced inflammation are worthy of further investigation.
- CBD does not appear to impair aerobic exercise performance and could, therefore, have utility within the sporting context.

## Introduction

Cannabidiol (CBD) is a non-intoxicating, plant-derived cannabinoid that has demonstrated considerable therapeutic potential [1]. CBD has well-established anticonvulsant effects [2–4], with the Food and Drug Administration (FDA) recently approving the oral CBD solution, *Epidiolex®*, for the treatment of intractable paediatric epilepsy [5]. Early-stage clinical trials have also demonstrated anxiolytic [6, 7] and antipsychotic [8, 9] effects. These effects are typically observed at oral doses of ~ 300–1500 mg CBD [10], although acute doses up to 6000 mg also appear to be safe and well-tolerated in humans, albeit with occasional mild side effects (e.g. diarrhoea, nausea, headache) [11, 12].

Alongside its emergent clinical use [13], interest in CBD has increased among general (non-clinical) populations [14, 15], including athletes [16]. While healthy individuals are usually ineligible to access regulated, prescription CBD (e.g. Epidiolex®), a wide range of low-dose “nutraceuticals” (e.g. oils, capsules, topicals and edibles typically containing between ~ 5 and 150 mg CBD·dose^−1^), including some products marketed specifically to athletes (e.g. cbdMD™, fourfivecbd™), are readily available over-the-counter in certain countries (e.g. UK, Canada, USA) [17]. Within the context of elite sports, the use of CBD has been further facilitated by its recent removal from the World Anti-Doping Agency’s “Prohibited List” [18]. In fact, 26% of British professional rugby players surveyed in a recent study (*n* = 517; 39% of those aged ≥ 28 years) reported either currently using, or having previously used, CBD [16]. The most common reasons for use were to enhance recovery (80%), improve sleep (78%), reduce anxiety (32%), and for “other” medical purposes (14%; e.g. concussion) [16].

Despite its growing popularity [16], only two interventional studies, both randomised, double-blind, placebo-controlled crossover trials, have so far investigated the effects of CBD on outcomes relevant to athletic performance. The first found no effect of CBD (150 mg·d^−1^ for 3 days) on non-invasive measures of muscle damage following eccentric exercise [19]. The second found CBD (60 mg; acute) decreased blood serum concentrations of creatine kinase and myoglobin, and increased one-repetition maximum back squat performance 72 h, but not 24 or 48 h following resistance exercise [20]. In any case, a recent review of preclinical studies and clinical trials (involving non-athlete populations) outlined the potential for CBD to exert anti-inflammatory, analgesic, anxiolytic and neuroprotective effects that could have utility in treating inflammatory pain (e.g. delayed onset muscle soreness, injuries), head injuries (e.g. concussion) and sports performance anxiety in athletes [21]. Of course, if CBD is to be used within the sporting context, it is important to understand how it influences key physiological and psychological responses during exercise, particularly given its complex pharmacology [22].

The current randomised, placebo-controlled exploratory pilot trial investigated the effects of acute, oral CBD treatment on physiological and psychological responses to submaximal and exhaustive running exercise in a small sample of endurance-trained males. It should be noted that, as a pilot study, this investigation was not designed nor formally powered to assess “effect” [23]. Rather, its intent was to gain a preliminary understanding of CBD’s effects on exercise physiology and to determine whether these are worthy of further investigation in larger, fully powered trials.

## Methods

This investigation was approved by the Sydney Local Health District’s Human Research Ethics Committee (2020/ETH00226), registered with the Australia and New Zealand Clinical Trials Registry (ACTRN12620000941965) and conducted at the Charles Perkins Centre—Royal Prince Alfred Hospital Clinic, Sydney, Australia, in accordance with Good Clinical Practice guidelines, the Declaration of Helsinki (1983) and local regulations.

### Study Design

Participants completed two treatment sessions involving the oral administration of CBD (300 mg) or a placebo in a randomised, double-blind, crossover design. Sessions were separated by a washout period ≥ 7 days on the basis that orally administered CBD (~ 300 mg) has been reported to have a half-life of ~ 24 h [24]. Individuals were instructed to maintain their usual diet and exercise patterns and avoid using cannabis and cannabinoids throughout their participation.

### Randomisation and Blinding

Participants were assigned to one of two possible treatment orders (CBD–Placebo or Placebo–CBD) in a 1:1 ratio by a blinded physician using a pre-populated randomisation schedule. This schedule was generated in two balanced blocks of four and one balanced block of two by an independent researcher, using an online random number generator (www.randomizer.org). Only this individual and pharmacists dispensing the treatments could access the randomisation schedule and neither had any contact with participants.

### Treatments

The investigational product (GD Cann®-C; GD Pharma Pty Ltd, Norwood, South Australia, Australia) was an oral formulation of synthetic CBD (100 mg·mL^−1^) in medium chain triglyceride (MCT) oil; the placebo was an equivalent volume of MCT oil, only. Neither product contained any other cannabinoids (including THC) or cannabis constituents (e.g., flavonoids, monoterpenes, sesquiterpenes) or differed noticeably in visual appearance or smell. Pharmacists dispensed the treatments into 5.0 mL syringes that carried no “treatment-identifying” information (e.g. letters, numbers) at the beginning of each session. The dose (300 mg CBD; 3.0 mL) was selected on the basis that it is the smallest amount to have, so far, reliably demonstrated clinically relevant effects in humans [11].

### Participant Characteristics

Healthy males aged between 18 and 45 years who had not used cannabis or cannabinoids in the previous three months (as confirmed by a negative urine drug screen [UDS]) and reported running an average of ≥ 40 km·wk^−1^ were recruited via word-of-mouth and using a general advertisement distributed to local running clubs. The full eligibility criteria are available in Additional file 1. A target sample size of *n* = 10 was selected with consideration for practical factors such as time, cost and resource allocation, rather than using formal statistical techniques, as this was an exploratory pilot study [25].

### Participant Screening

Volunteers completed a short telephone interview before scheduling a face-to-face screening visit, during which they were informed of the study requirements and risks and provided written informed consent. Eligibility was then assessed by an investigator and physician as per the criteria in Additional file 1. Finally, eligible participants performed an incremental treadmill test to determine maximal oxygen consumption (V̇O_2max_) and to become familiar with trial procedures. The protocol used during the initial V̇O_2max_ test was identical to that used during the experiment (“Incremental Exercise (RUN 2)” section), except respiratory gases were sampled continuously throughout. The average rate of oxygen consumption (V̇O_2_) over the final 30 s of each (completed) increment was also calculated, and the linear relationship between V̇O_2_ and treadmill gradient determined, to set the exercise intensity during subsequent sessions.

### Experimental Procedures

**Fig. 1:**
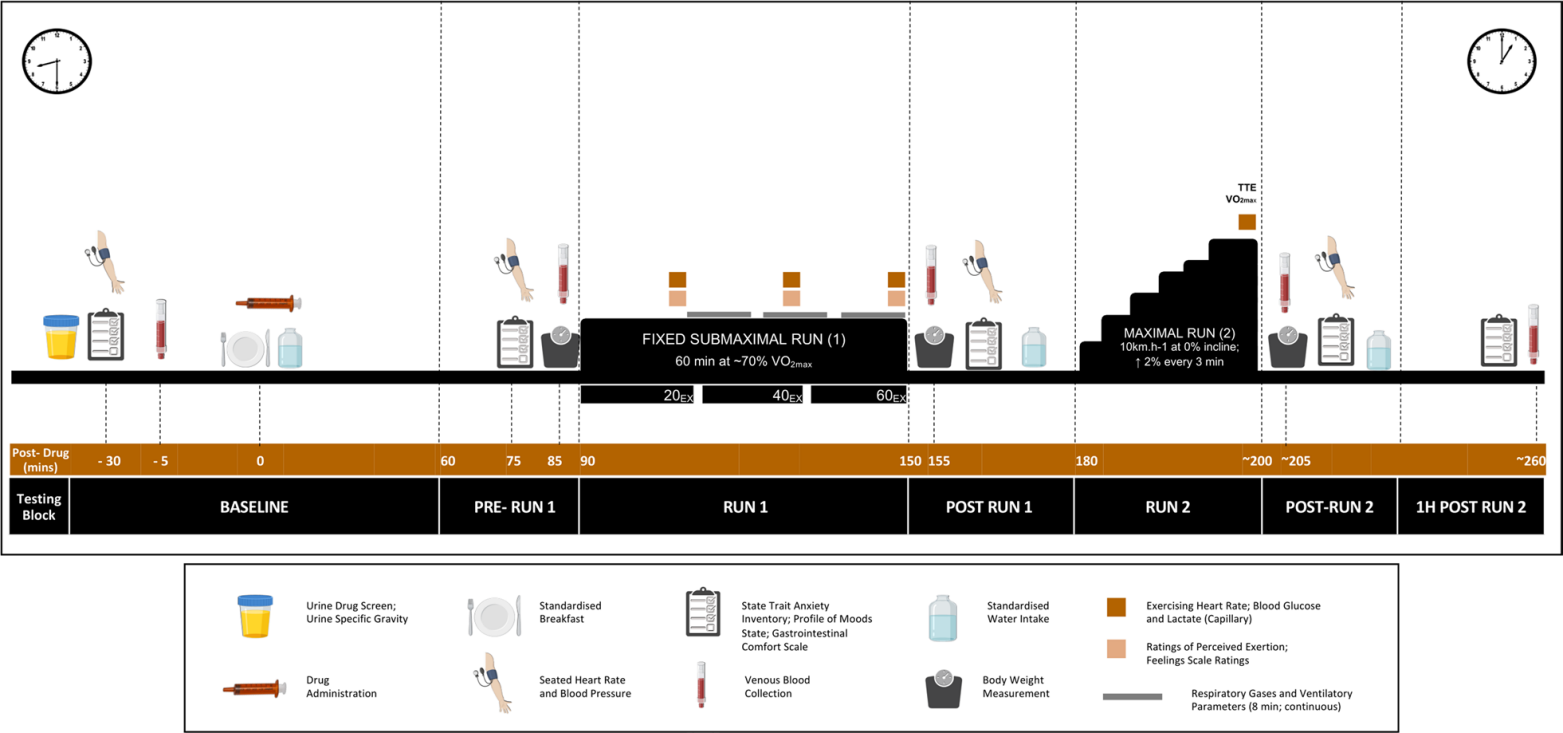
A schematic representation of experimental sessions. TTE: time to exhaustion; VO_2max_: aerobic capacity

The experimental procedures and timelines are summarised in Fig. 1.

#### Standardisation Procedures

Participants were instructed to: (1) abstain from alcohol (> 24 h) and caffeine (> 12 h); (2) avoid moderate to strenuous exercise (> 24 h); (3) avoid anti-inflammatory medication (> 24 h); (4) keep a diet record (24 h); (5) consume a pre-packaged standardised evening meal (~ 60 kJ·kg^−1^; ~ 2.0 g·kg^−1^ carbohydrate [CHO]); (6) fast overnight (~ 10 h); (7) spend ≥ 8 h in bed; and (8) collect a first-morning urine sample and consume 500 mL of water before presenting to the laboratory. Individuals received a copy of their pre-trial diet record after the first session and were instructed to replicate their behaviour before their second visit.

#### Trial Procedures

Participants arrived at the laboratory in a fasted state at ~ 08:30 AM on the morning of each treatment session where they verbally acknowledged compliance to the standardisation procedures and completed a UDS to verify cannabis abstinence (Drug Check® NxStep OnSite Urine Drug Test). The first-morning urine sample was also analysed to determine urine-specific gravity (U_SG_; Palette Digital Refractometer, ATAGO, USA). If U_SG_ was > 1.024, likely indicating hypohydration [26], a second sample was collected and analysed (all had U_SG_ values ≤ 1.024).

Each session involved seven consecutive blocks of testing: Baseline (pre-treatment), Pre-RUN 1 (+ 60–90 min post-treatment), RUN 1 (+ 90–150 min), Post-RUN 1 (+ 155–170 min), RUN 2 (+ 180– approx. 200 min), Post-RUN 2 (approx. + 205–220 min) and 1 h Post-RUN 2 (approx. + 220–260 min). The assessments completed during each block are summarised in Fig. 1 and detailed.

Baseline tests consisted of resting heart rate (HR) and blood pressure (BP) measurements, the completion of the short-form State-Trait Anxiety Inventory (STAI-S), the short-form Profile of Mood States (POMS), and gastrointestinal (GI) comfort questionnaires, and sampling of venous blood. Thereafter, participants received a standardised breakfast consisting of raisin toast (two slices) (TipTop®) and up to 30 g of raspberry jam (Cottee’s®), 20 g of margarine (Flora® Proactive Original) and 500 mL of water (as preferred); individual intakes were recorded and replicated across sessions. Treatments were self-administered (via oral ingestion) following the consumption of breakfast and a high-strength mint (Fisherman’s Friend®) designed to mask any differences in flavour [27]. Exercise was performed on a motorised treadmill (Trackmaster® TMX428CP) in a thermoneutral laboratory as described in “Submaximal Exercise (“RUN 1”)” section and “Incremental Exercise (“RUN 2”)” section. Participants were not permitted to consume fluid during exercise but received up to 500 mL of water on completion of the Post-RUN 1 and 2 assessments; again, individual intakes were recorded and replicated across sessions. At the end of each treatment session, participants completed an adverse event (AE) checklist and indicated which treatment they thought they had received and their level of confidence in their guess (on a 4-point Likert scale; 1 =“not at all” to 4 =“extremely”).

#### Submaximal Exercise (“RUN 1”)

Submaximal exercise (RUN 1) began 90 min post-treatment. Participants ran for 60 min at a fixed speed (10 km·h^−1^) and individualised gradient designed to elicit an intensity of 70% V̇O_2max_. Respiratory gases were sampled continuously between 24–32 (24_EX_), 37–45 (37_EX_) and 50–58 (50_EX_) min of exercise. Measures were collected at precisely the same time to minimise any influence of metabolic drift [28]. HR (Polar H10 HR Sensor), ratings of perceived exertion (RPE) on the Borg scale (6–20) [29], ratings of affect (i.e. pleasure–displeasure) on the Feelings Scale (–5 =“feeling very bad” to + 5 =“feeling very good”) [30], and finger prick blood lactate (BL) and glucose (BG) concentrations (in singlicate) (Edge® Blood Lactate Monitoring System; Accu-Check® Performa Meter) were also measured at 20 (20_EX_), 40 (40_EX_) and 60 (60_EX_) minutes.

#### Incremental Exercise (“RUN 2”)

The incremental exercise test (RUN 2) began 180 min post-treatment. The test was completed at a fixed speed (10 km·h^−1^), with the gradient commencing at 0% and increasing by 2% every 3 min until volitional exhaustion. Participants did not receive feedback on elapsed time or encouragement during the test. Respiratory gases were sampled during the final ~ 5 min of exercise (i.e. from the point at which HR exceeded ~ 90% HR_max_, as determined during the initial V̇O_2max_ test). Time to exhaustion (TTE) and maximum HR attained (HR_max_) were recorded.

### Data Collection

#### Respiratory Gases

Respiratory gases were sampled using an Ultima PFX® pulmonary function system (MGC Diagnostics®) with a PreVent™ flow pneumotach (MCG Diagnostics®) and mouthpiece. The flow transducer and gas analysers were calibrated daily. Breath-by-breath measurements of V̇O_2_, expired CO_2_ (V̇CO_2_), respiratory exchange ratio (RER), respiratory rate (RR), tidal volume (V_T_) and minute ventilation (V_E_) were obtained and averaged across each collection period. V̇O_2max_ was taken as the highest average V̇O_2_ attained over 30 s; V̇CO_2max_ and RER_max_ were taken as the average of the aforementioned period.

#### Sweat Loss

Nude body weight (BW) was measured Pre- and Post-RUN 1 and Post-RUN 2 to estimate sweat loss. Water intake and urinary losses were measured and factored into all estimations.

#### Resting Blood Pressure (BP)

Seated BP was measured at Baseline, Pre- and Post-RUN 1, Post-RUN 2 and 1 h Post-RUN 2 using an automated sphygmomanometer (OMRON®, M2 Basic). Measurements were taken in duplicate or triplicate if systolic BP values differed by > 15 mmHg and then averaged prior to analysis [31].

#### Gastrointestinal (GI) Comfort

GI comfort (“Abdominal Pain”, “Nausea”, “Heartburn”, “Regurgitation”, “Belching”, “Bloating” and “Flatulence”) was measured at Baseline, Pre- and Post-RUN 1, Post-RUN 2 and 1 h Post-RUN 2 using 100 mm visual analogue scales (VAS) where 0 mm represented “not at all” and 100 mm, “extremely”.

#### Subjective Feelings

State anxiety and mood were measured at Baseline, Pre- and Post-RUN 1, Post-RUN 2 and 1 h Post-RUN 2 using the short-form STAI-S [32] and short-form POMS [33] as described in Additional file 1.

#### Blood Sampling and Biomarker Analyses

Blood was collected into 10 mL pre-treated EDTA vacutainers and 6 mL serum vacutainers (VACUETTE®, Greiner Bio-One, Kremsmünster, Austria) at Baseline, Pre-RUN 1 (plasma only), Post-RUN 1, Post-RUN 2 and 1 h Post-RUN 2 via a cannula inserted into an antecubital forearm vein. Samples were centrifuged at 2500 RCF for 15 min (4 °C) after the serum sample had clotted (approx. 15 min). Aliquots of supernatant were stored at − 80 °C until analysis.

Plasma was thawed and analysed using ultra-high performance liquid tandem mass spectrometry (UHPLC-MS/MS) and previously validated methods [34]. Target analytes were CBD, 7-COOH-CBD, 7-OH-CBD, 6-OH-CBD, THC, 11-OH-THC and 11-COOH-THC. The maximum plasma CBD concentration (*C*_max_) and time to *C*_max_ (*T*_max_) were estimated for each individual participant; specifically, *C*_max_ was taken as the highest concentration of CBD measured in plasma and *T*_max_ was taken as the timepoint at which *C*_max_ occurred. Plasma anandamide (AEA) concentrations were also determined using UHPLC-MS/MS (see Additional file 1 for methods).

Serum samples were thawed and analysed to determine circulating interleukin (IL)-1β, tumour necrosis factor-α (TNF-α), myoglobin (Mb), creatine kinase (CK) and claudin-3 (NBP2-75,328; Novus Biologicals, Centennial, USA) concentrations using commercially available enzyme-linked immunosorbent assay (ELISA) kits (see Additional file 1 for methods). Circulating lipopolysaccharide (LPS) was determined using the limulus amebocyte lysate (LAL) chromogenic endpoint assay (see Additional file 1 for methods).

#### Next-day Sleep Quality and Muscle Soreness

Sleep quality (–5 = “very poor” to + 5 =“very good”) and muscle soreness (0 = “not at all” to 10 = “extremely”) were measured the morning following each treatment session using Likert scales.

### Statistical Analyses

Being exploratory and pilot in nature, the current study was not designed nor formally powered to assess “effect” [25]. Rather, the intent was to gain a preliminary understanding of CBD’s effects on exercise physiology and determine whether these are worthy of further investigation in a larger, fully powered trial. As such, data analysis involved the determination of effect sizes and confidence intervals (CIs) (23). Cohen’s *d*_z_ effect sizes (chosen to facilitate future sample size calculations) were calculated by standardising the mean difference between each placebo and intervention outcome measure against the SD of change (SD_Δ_) [35]. The standard error (SE) was then derived using the Hedges & Olkin approximation adapted for a repeated measures design [36, 37]:

1 $${\text{SE}}_{{\text{d}}} = \sqrt {\left( { \frac{1}{n} + \frac{{d^{2} }}{2n} } \right) \times 2 \times \left( {1 - R} \right)}$$

where SE_d_ is the SE of Cohen’s *d*, *d* is Cohen’s *d*_z_, *n* is the sample size and *R* is the correlation coefficient. SE_d_ values were then divided by a factor of $\sqrt {2 \left( {1 - R} \right)}$ to derive the SE of Cohen’s *d*_z_ specifically [36, 38], and 85% CIs were derived via standard methods [39]. Lee, Whitehead, Jacques and Julious [23] recommend using 75% or 85% CIs rather than the common 95% threshold to investigate pilot data. CBD’s effects were then interpreted as follows: “*uncertain”* if the 85% CI included zero and ± 0.5; “*unlikely affected*” if the 85% CI included zero but not ± 0.5; and “*possibly affected*” (i.e. worthy of further investigation) if the 85% CI included ± 0.5 but not zero. Thresholds (± 0.5) were selected on the basis that they represent a “moderate” Cohen’s *d*_z_ effect [39].

Statistical analyses were performed using SPSS Statistics, Version 26.0 (IBM Corp. 2019, Armonk, N.Y., USA). Treatment $\times$ Time repeated-measures analyses of variance (ANOVA) was used to investigate time effects on blood biomarkers (only); that is, on outcomes where an effect of CBD is predicated on an exercise-induced change. All measures were normally distributed (Shapiro–Wilk test, *p*’s > 0.05). Where assumptions of sphericity (Mauchly’s test) were violated, the Greenhouse–Geisser correction was applied. Paired *t*-tests were used to conduct post hoc comparisons on significant time effects (at the least significant difference) and compare standardisation outcomes across treatment sessions. Statistical significance was accepted as *p* < 0.05. Data are reported as Mean ± SD, unless otherwise stated.

## Results

### Participant Characteristics and Standardisation Procedures

**Fig. 2:**
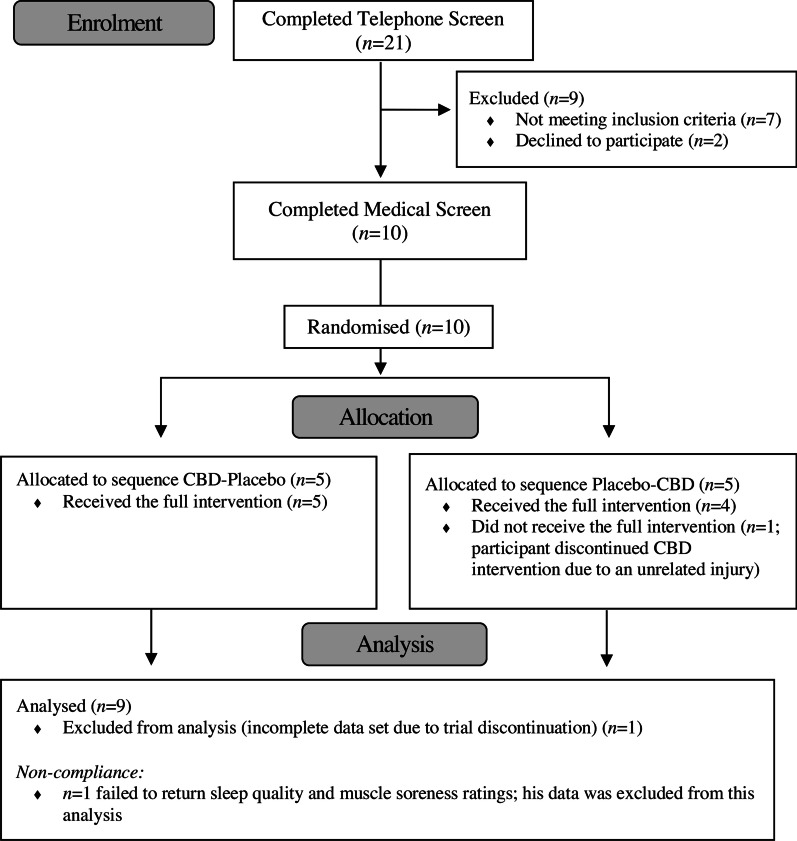
Consolidated Standards of Reporting Trials (CONSORT) diagram. Recruitment was completed when the target sample size of *n* = 10 participants had been randomised

Ten participants were recruited and randomised between August 2020 and October 2020 (Fig. 2). One participant withdrew during their second session (< 5 min into RUN 1) due to an injury sustained elsewhere and was removed from the final sample. The characteristics of the nine remaining participants are summarised in Table 1. All participants acknowledged compliance with the pre-trial procedures and successfully replicated the same experimental protocol at both sessions. Baseline BW (Placebo = 70.5 ± 5.4 kg, CBD = 70.7 ± 5.5 kg, *t*(8) = 0.984,* p* = 0.354) and U_SG_ (Placebo = 1.015 ± 0.008, CBD = 1.015 ± 0.009, *t*(7) = 0.025, *p* = 0.980) as well as the laboratory temperature (Placebo = 21.5 ± 0.44 °C, CBD = 21.2 ± 0.3 °C, *t*(8) = 1.538, *p* = 0.163) and humidity (Placebo = 54.8 ± 8.6%, CBD = 56.7 ± 7.4%, *t*(8) = 0.634, *p* = 0.544) were similar across sessions. Participants consumed 4444 ± 390 kJ and 140 ± 9 g CHO for dinner the night prior to each session. Participants consumed 1291 ± 238 kJ, 50.2 ± 8.6 g CHO and 389 ± 220 mL of water during breakfast on the day of each session. Water consumption Post-RUN 1 and 2 was 343 ± 109 mL and 425 ± 153 mL, respectively.

**Table 1:** Participant characteristics (*n* = 9)

Age (y)	33 (8) (24–43)
Weight (kg)	71.2 ± 5.3 (63.1–78.0)
Height (cm)	181 ± 7 (168–190)
VO_2max,_ (mL·kg^−1^·min^−1^)	57.4 ± 4.0 (53.1–65.0)
Running distance (km·wk^−1^)	63 ± 21 (40–100)
Time since reported last cannabis use (*n*)	
3–12 months	0
1–2 years	2
2–4 years	3
> 4 years	2
No prior use	2
Lifetime cannabis exposure (*n*)	
≤ 10 uses	5
> 10 uses	2
No prior use	2
Lifetime CBD exposure (*n*)	
≤ 10 uses	0
> 10 uses	1^a^
No prior use	8

VO_2max_: Aerobic capacity. Values are mean ± SD (range), median (IQR) (range) and number of participants (*n*), as appropriate

^a^1–2 y since last use

### Plasma Cannabinoid and Endocannabinoid Concentrations

**Fig. 3:**
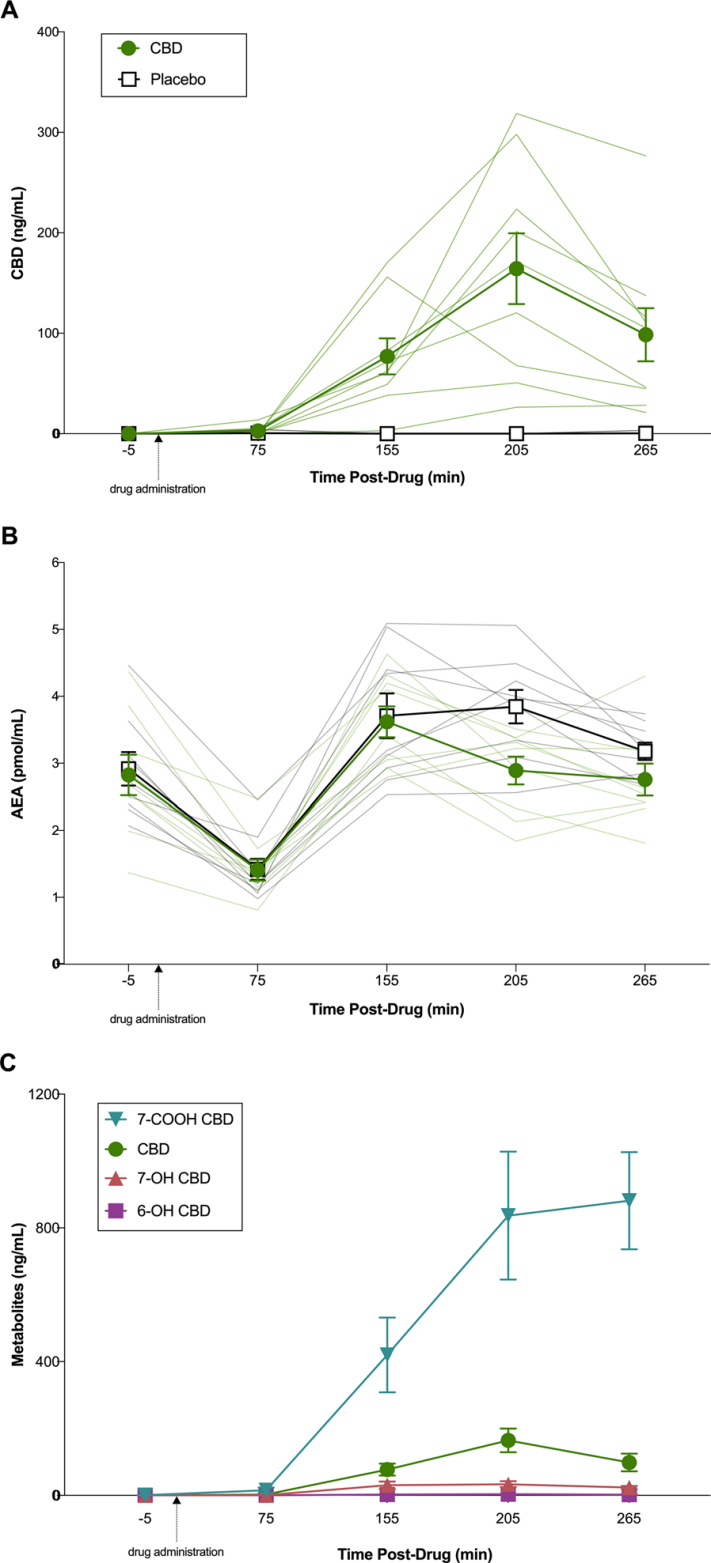
Plasma concentration over time profiles for cannabidiol (CBD) and anandamide (AEA) during acute CBD (300 mg) or placebo treatment. **A** CBD concentrations during CBD (green circle) and placebo (white square) treatment. **B** AEA concentrations during CBD (green circle) and placebo (white square) treatment. **C**. CBD metabolite concentrations during CBD treatment only. Venous blood was collected—5- (Baseline), 75- (Pre-RUN 1), 155- (Post-RUN 1), 205- (Post-RUN 2) and 265- (1 h Post-RUN 2) min post-drug administration. Dark lines represent mean ± SEM (*n* = 9) and light lines represent individual participant values

Plasma CBD, 7-COOH-CBD, 7-OH-CBD and 6-OH-CBD concentrations are displayed in Figs. 3a, c. *C*_max_ and *T*_max_ were estimated as 174 ± 100 ng∙mL^−1^ and 206 ± 25 min, respectively. Although sessions were separated by an average of 8.6 ± 2.4 days (and a minimum of 7 days), all five of the participants who completed the CBD session before the placebo session had detectable levels of 7-COOH-CBD in plasma at Baseline on their placebo session (≤ 19.1 ng·mL^−1^); two also had very low concentrations of CBD (≤ 0.8 ng·mL^−1^). THC, 11-OH-THC and 11-COOH-THC were not detected in any samples.

Plasma AEA concentrations are displayed in Fig. 3b. AEA showed a significant effect of Time (AEA: *F*_1,8_ = 37.46, *p* < 0.001, ηp^2^ = 0.83) with post hoc comparisons revealing lower plasma AEA concentrations Pre-RUN 1 compared to Baseline (*p* < 0.001) and higher AEA concentrations Post-RUN 1, Post-RUN 2 and 1 h Post-RUN 2, compared to Baseline and Pre-RUN 1 (*p*’s < 0.05). Plasma AEA concentrations appeared *possibly* reduced Post-RUN 2 under the CBD treatment, relative to placebo (Cohen’s *d*_z_ = − 1.492, 85% CI’s = − 2.190, − 0.794). The effect of CBD at all other timepoints was *uncertain.*

### Submaximal Exercise (“RUN 1”)

**Fig. 4:**
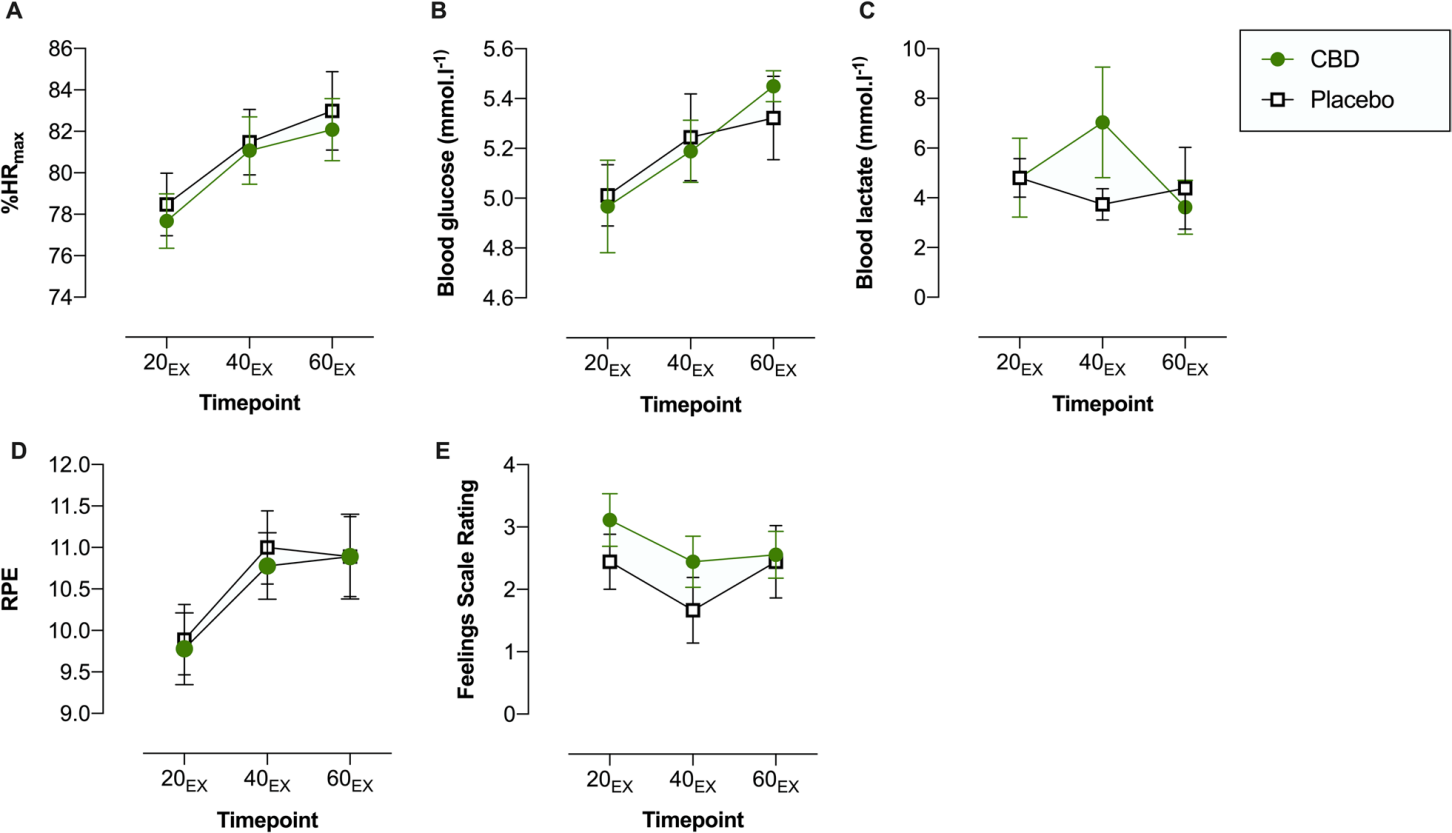
Physiological and subjective outcomes assessed at 20- (20_EX_), 40- (40_EX_), and 60- (60_EX_) minutes of fixed submaximal (~ 70% VO_2max_) exercise (RUN 1): **A** Heart Rate (expressed as age-predicted %HR_max_); **B** Blood Glucose; **C** Blood Lactate; **D** Ratings of Perceived Exertion (RPE); **E** Feelings Scale (FS) Ratings. Values are mean ± SEM for placebo (white squares) and CBD (green circles) (*n* = 9)

**Fig. 5:**
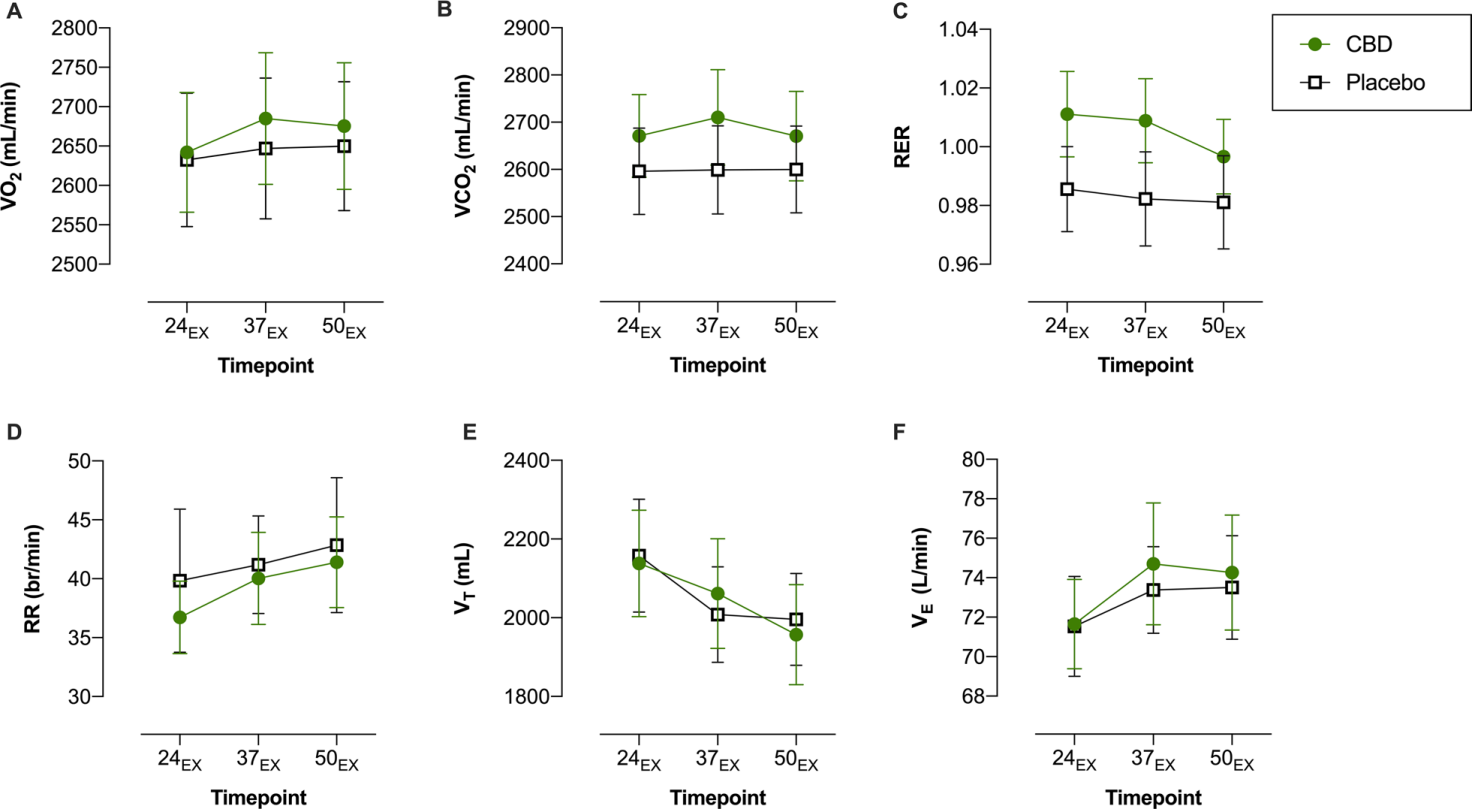
Respiratory gas measures assessed during fixed submaximal (~ 70%VO_2max_) exercise (RUN 1). **A** Oxygen Consumption (VO_2_); **B** Carbon Dioxide Production (V̇CO_2_); **C** Respiratory Exchange Ratio (RER); **D** Respiratory Rate (RR); **E** Tidal Volume (V_T_) and **F** Minute Ventilation (*V*_E_). Breath-by-breath registrations of respiratory gases were collected at three intervals (24–32 (24_EX_), 37–45 (37_EX_) and 50–58 (50_EX_) minutes) during RUN 1. These measures were averaged across each 8-min collection period prior to analysis. Values are mean ± SEM for placebo (white squares) and CBD (green circles) (*n* = 9)

The outcomes measured during RUN 1 are displayed in Figs. 4 and 5; Cohen’s d_z_ effect sizes are presented in Fig. 7. The effects of CBD on HR, blood glucose, RPE, RER, RR, *V*_E_, *V*_T_ and estimated fluid loss were *uncertain*. Blood lactate at 40_EX_ (Placebo = 3.73 ± 1.91, CBD = 7.03 ± 6.68), V̇O_2_ at 37_EX_ (Placebo = 2647 ± 268 mL·min^-1^, CBD = 2685 ± 251 mL·min^-1^), V̇CO_2_ at 24_EX_ (Placebo = 2596 ± 275 mL·min^-1^, CBD = 2671 ± 263 mL·min^-1^) and 37_EX_ (Placebo = 2599 ± 281 mL·min^-1^, CBD = 2710 ± 304 mL·min^-1^) and ratings of pleasure on the Feelings Scale at 20_EX_ (Placebo = 2.4 ± 1.3, CBD = 3.1 ± 1.3) and 40_EX_ (Placebo = 1.7 ± 1.6, CBD = 2.4 ± 1.2) all appeared *possibly* elevated with the CBD treatment, relative to placebo_._ Effects were *uncertain* at all other timepoints.

### Incremental Exercise (“RUN 2”)

**Fig. 6:**
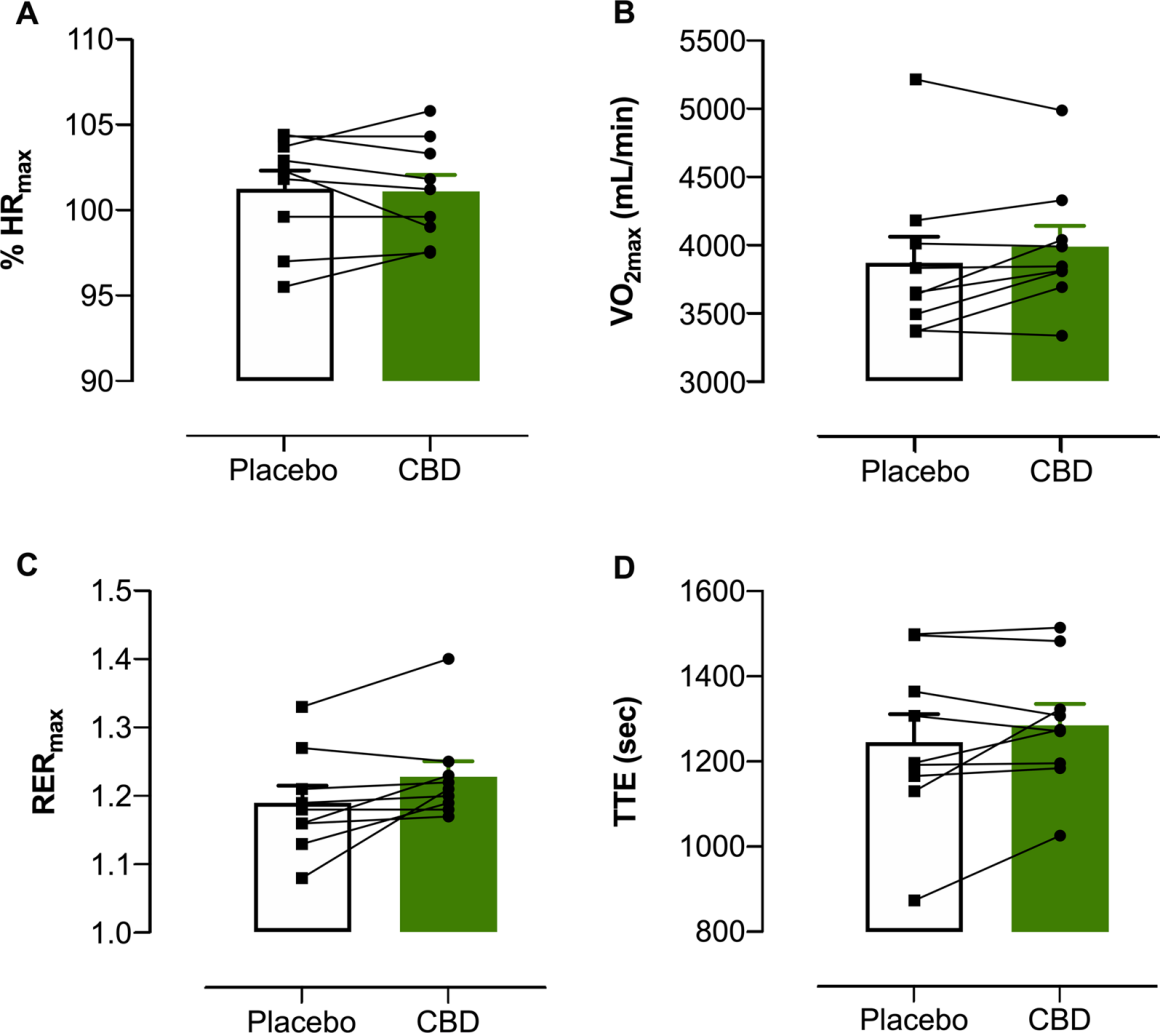
Outcomes measured during the incremental exercise test to volitional exhaustion (RUN 2). **A** Maximum Heart Rate (expressed as % HR_max_); **B** Maximum Oxygen Consumption (VO_2max_); **C** Respiratory Exchange Ratio (RER) and **D** Time to Exhaustion (TTE). Values are mean ± SEM for placebo (white) and CBD (green); each line represents one individual participant (*n* = 9)

**Fig. 7:**
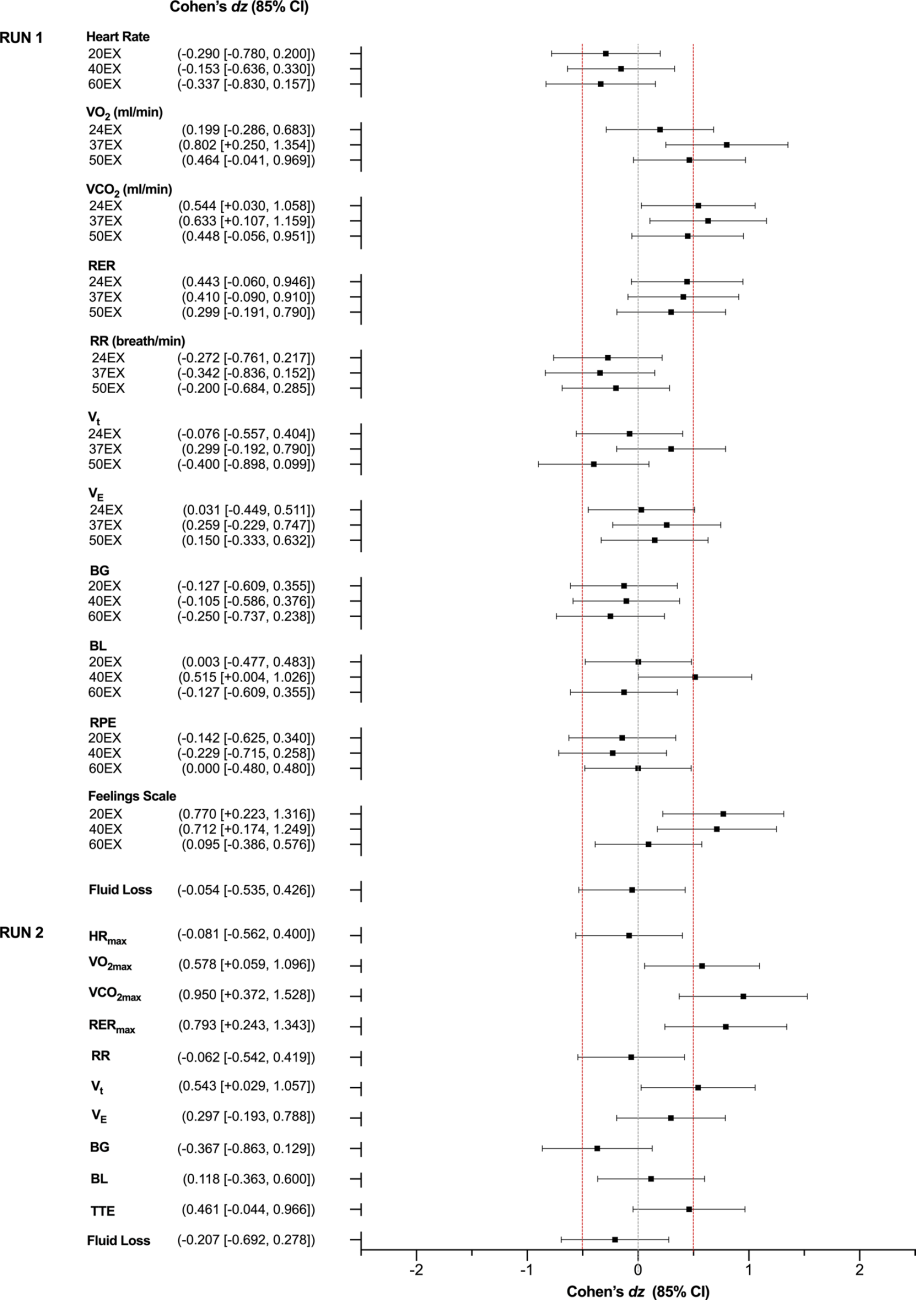
Key physiological and psychological outcomes assessed during the submaximal (RUN 1) and exhaustive (RUN 2) exercise tasks. Values are Cohen’s *d*_z_ effect sizes and 85% confidence intervals (CI). BG: Blood Glucose; BL: Blood Lactate; EX: Minutes of Exercise; RER_max_: Maximum Respiratory Exchange Ratio; RPE: Ratings of Perceived Exertion; RR: Respiratory Rate; TTE: Time to Exhaustion; V_E_: Minute Ventilation; V_t_: Tidal Volume; V̇CO_2_: Carbon Dioxide Expiration VO_2_: Oxygen Consumption; V̇CO_2max_: Maximal V̇CO_2_; VO_2max_: Maximal VO_2_. Positive effect sizes indicate an increase for CBD relative to placebo and negative effect sizes, a decrease. Confidence intervals were calculated using standard methods (*n* = 9)

The outcomes measured during RUN 2 are displayed in Fig. 6; Cohen’s *d*_z_ effect sizes are presented in Fig. 7. The effects of CBD on HR_max_, TTE, blood lactate, blood glucose, RR and *V*_E_ were *uncertain*. V̇O_2max_ (Placebo = 3868 ± 577 mL⋅min^-1^, CBD = 3987 ± 462 mL⋅min^-1^), V̇CO_2max_, (Placebo = 4594 ± 704 mL⋅min^-1^, CBD = 4871 ± 524 mL⋅min^-1^), RER_max_ (Placebo = 1.19 ± 0.07, CBD = 1.23 ± 0.07) and *V*_t_ (Placebo = 2862 ± 680 mL, CBD = 2957 ± 608 mL) all appeared *possibly* elevated with the CBD treatment, relative to placebo. Neither TTE (Placebo = 1246 ± 197 s, CBD = 1286 ± 150 s, *t*(8) = 0.528, *p* = 0.612) nor V̇O_2max_ (Placebo = 3868 ± 577 mL·min^−1^, CBD = 3987 ± 462 mL·min^−1^, *t*(8) = 0.067, *p* = 0.948) demonstrated significant trial order effects.

### Serum Biomarkers

Serum TNF-α, IL-1β, IL-6, Mb, CK, LPS and Claudin-3 concentrations are displayed in Additional file 1.

TNF-α, IL-6, Mb, CK and LPS showed significant effects of Time (TNF-α: *F*_1,8_ = 3.19, *p* = 0.045, ηp^2^ = 0.31; IL-6: *F*_1,8_ = 15.06, *p* < 0.001, ηp^2^ = 0.65; Mb: *F*_1,8_ = 10.36, *p* < 0.001, ηp^2^ = 0.56; CK: *F*_1,8_ = 21.00, *p* = 0.001, ηp^2^ = 0.72; LPS: *F*_1,8_ = 4.34, *p* = 0.046, ηp^2^ = 0.35). Post hoc comparisons revealed higher serum IL-6 and Mb concentrations Post-RUN 1, Post-RUN 2 and 1 h Post-RUN 2 compared to Baseline (*p’s* < 0.05). LPS concentrations were also higher Post-RUN 2 (*p* = 0.021) and 1 h Post-RUN 2 (*p* = 0.020) compared to Baseline. TNF-α concentrations were higher Post-RUN 2 (*p* = 0.016) and 1 h Post-RUN 2 (*p* = 0.026) than Post-RUN 1.

**Fig. 8:**
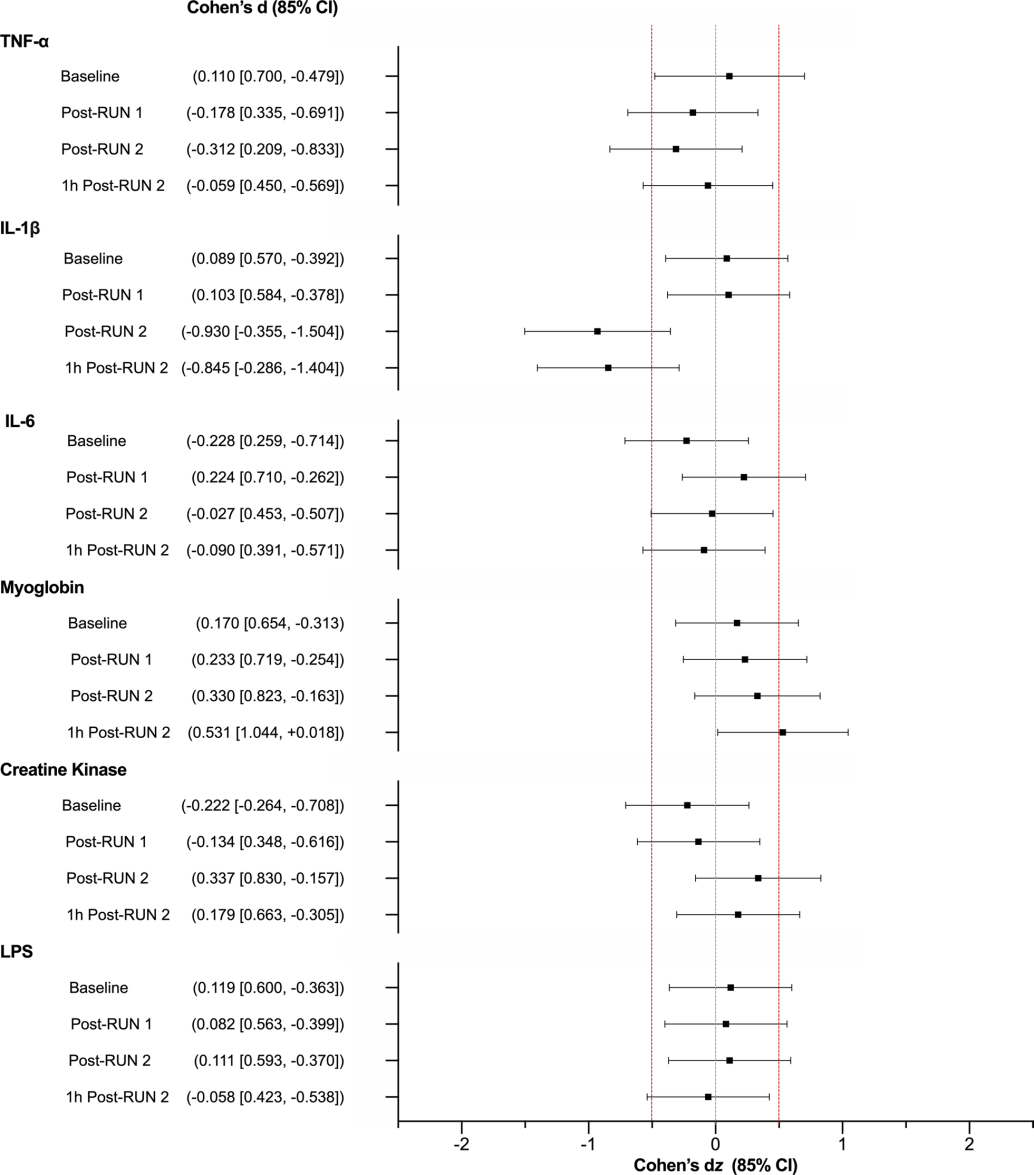
Biomarkers of exercise-induced inflammation assessed at Baseline, Post-RUN 1, Post-RUN 2 and 1 h Post-RUN 2. Values are Cohen’s *d*_z_ effect sizes and 85% confidence intervals (CI) TNF-α: tumour necrosis factor-α; IL-1β: interleukin-1β; IL-6: interleukin-6; LPS: liposaccharide binding protein. Positive effect sizes indicate an increase for CBD relative to placebo and negative effect sizes, a decrease. Confidence intervals were calculated using standard methods (*n* = 9)

Cohen’s d_z_ effect sizes were calculated for those biomarkers wherein an exercise-induced change was observed (“Statistical analysis” section). These results are presented in Fig. 8. The effects of CBD on TNF-α, IL-6, CK and LPS were *uncertain*. With CBD treatment: IL-1β concentrations appeared *possibly* decreased Post-RUN 2 (Placebo = 0.09 ± 0.06 pg·mL, CBD = 0.05 ± 0.04 pg·mL) and 1-h Post-RUN 2 (Placebo = 0.06 ± 0.05 pg·mL, CBD = 0.04 ± 0.02 pg·mL), while Mb concentrations appeared *possibly* increased 1 h Post-RUN 2 with CBD relative to placebo (Placebo = 748 ± 124 ng·mL, CBD = 845 ± 183 ng·mL). Effects were *uncertain* at all other timepoints.

### Gastrointestinal (GI) Comfort

GI comfort VAS ratings are presented in Additional file 1. These outcomes were not subjected to further analysis as participants only ever reported low levels of GI discomfort (< 10 mm) (except one individual, who rated all symptoms > 10 mm on both sessions).

### Subjective Feelings

**Fig. 9:**
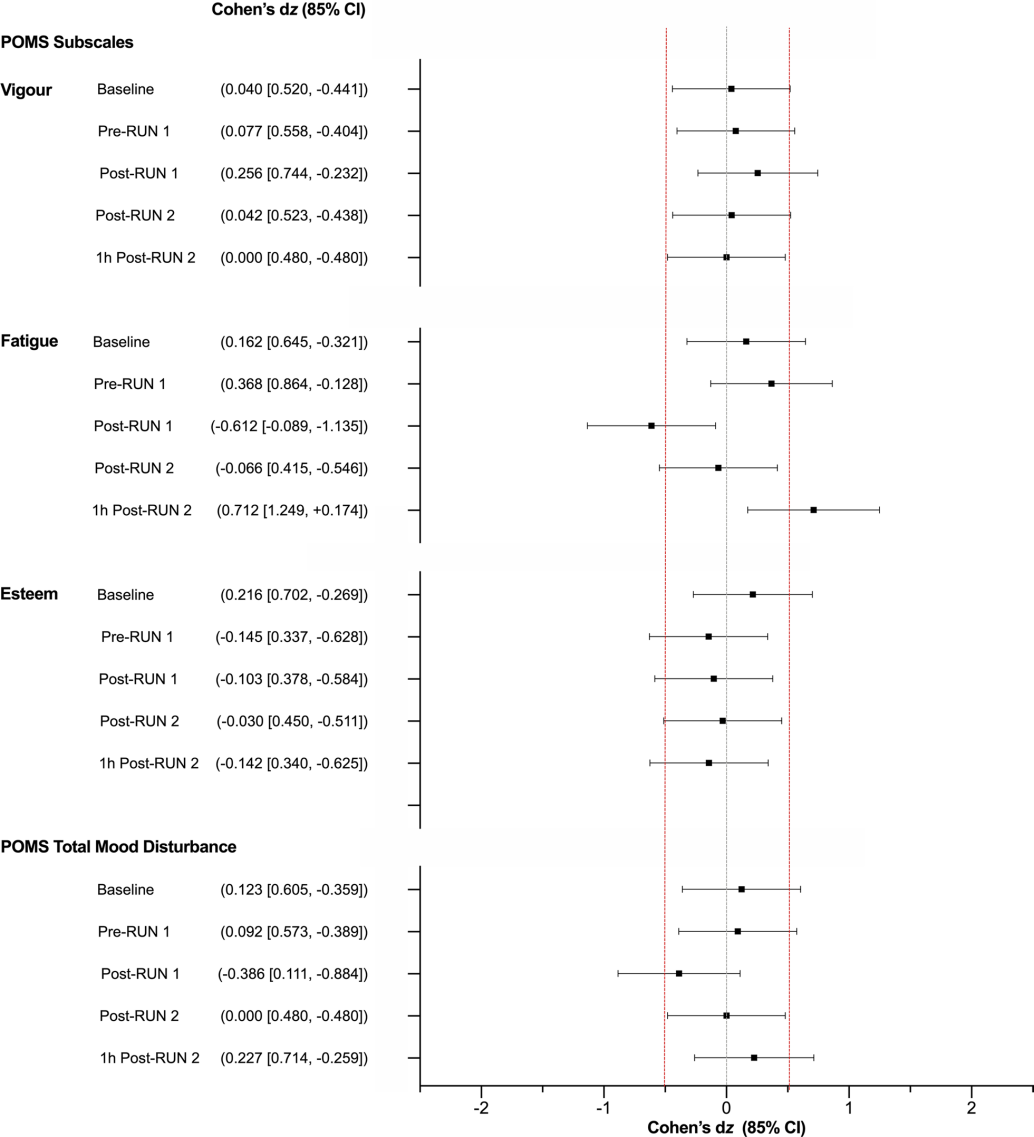
Profile of Mood States (POMS) Ratings as measured at Baseline, Pre- and Post-RUN 1, Post-RUN 2 and 1 h Post-RUN 2. Total mood disturbance (TMD) was calculated by subtracting the sum of positive emotional states (“Vigour” and “Esteem”) from negative states (“Tension”, “Depression”, “Fatigue”, “Confusion” and “Anger”) and adding 100. Values are Cohen’s *d*_z_ effect sizes and 85% confidence intervals (CI) positive effect sizes indicate an increase for CBD relative to placebo and negative effect sizes, a decrease. Confidence intervals were calculated using standard methods (*n* = 9)

STAI-S and POMS scores are presented in Additional file 1. The STAI-S and the “Tension”, “Depression”, “Anger” and “Confusion” sub-scales of the POMS were not subjected to further analysis as participants only ever recorded low scores on these scales. Cohen’s *d*_z_ effect sizes for the “Vigour”, “Esteem” and “Fatigue” subscales are presented in Fig. 9. The effect of CBD on “Vigour” and “Esteem” was *uncertain*, while “Fatigue” appeared *possibly* reduced Post-RUN 1 (Placebo = 2.0 ± 1.9, CBD = 1.1 ± 1.7) and *possibly* elevated 1 h Post-RUN 2 with CBD treatment, relative to placebo (Placebo = 1.3 ± 1.7, CBD = 2.1 ± 2.1). Effects were *uncertain* at all other timepoints.

### Resting Heart Rate (HR) and Blood Pressure (BP)

**Fig. 10:**
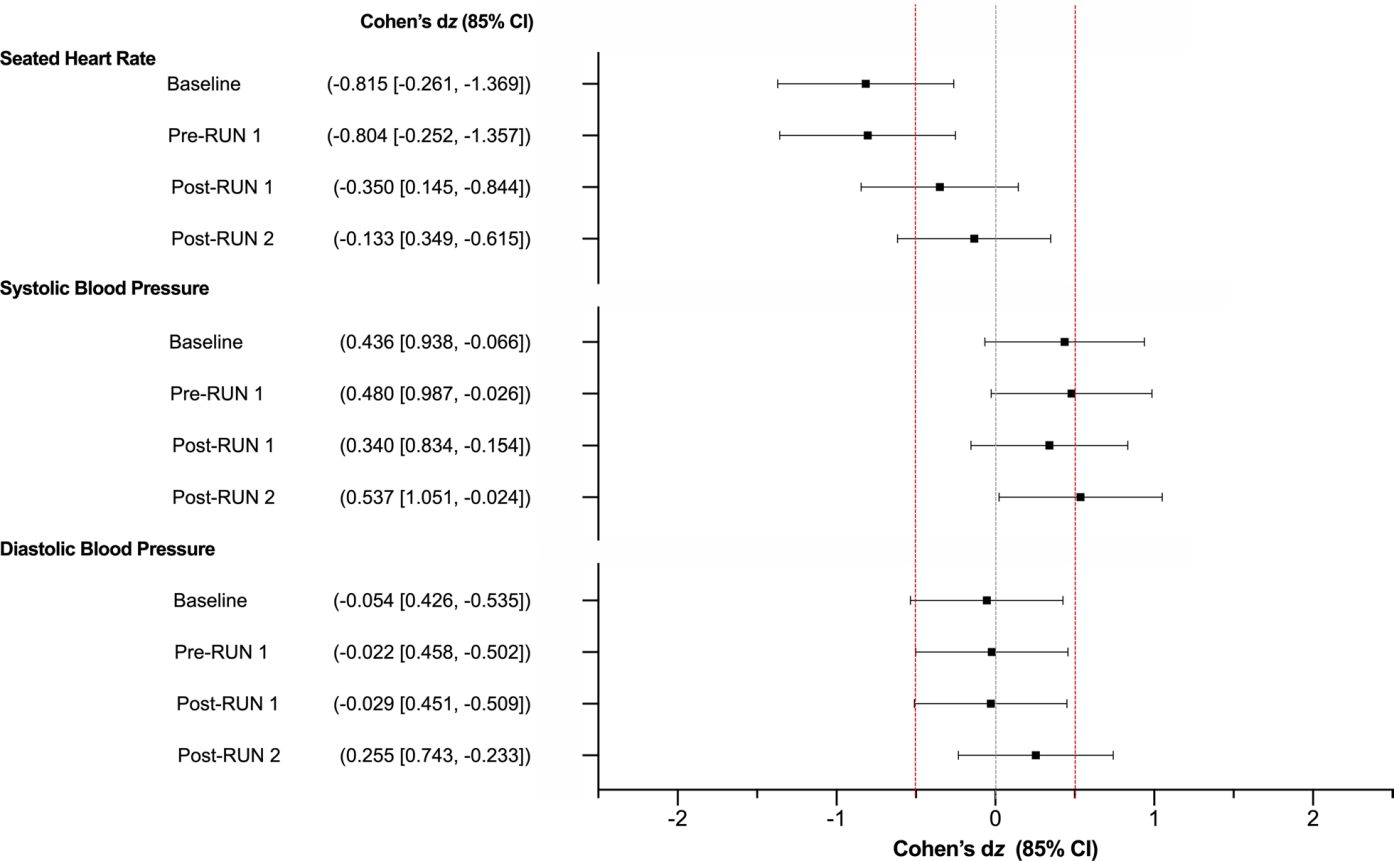
Seated heart rate and blood pressure as measured at Baseline, Pre-RUN 1, Post-RUN 1 and Post-RUN 2. Values are Cohen’s *d*_z_ effect sizes and 85% confidence intervals (CI). Positive effect sizes indicate an increase for CBD relative to placebo and negative effect sizes, a decrease. Confidence intervals were calculated using standard methods (*n* = 9)

Resting HR and BP measurements are presented in Additional file 1; Cohen’s d_z_ effect sizes are presented in Fig. 10. The effect of CBD on systolic and diastolic BP was *uncertain*. HR appeared *possibly *reduced with CBD treatment relative to placebo Pre-RUN 1 (Placebo = 55 ± 4 bpm, CBD = 51 ± 6 bpm), although this difference also appeared to be present at Baseline. Effects were *uncertain* at all other timepoints.

### Sleep Quality and Next-Day Muscle Soreness

The effect of CBD on next-day subjective muscle soreness (Placebo = 1.8 ± 0.3, CBD = 1.6 ± 0.3; Cohen’s *d*_z_ = − 0.195, 85% CI’s: − 0.709, 0.319) and sleep quality (Placebo = 3.3 ± 0.2, CBD = 3.4 ± 0.7; Cohen’s *d*_z_ = 0.195, 85% CI’s: –0.319, 0.709) was *uncertain*. (Nb. These analyses were performed at *n* = 8 as one participant failed to return his ratings after one session).

### Participant Expectations and Blinding

Prior to participation, most participants (*n* = 6) believed that CBD would have a “slightly positive” effect on their endurance exercise performance; one believed it would have a “slightly negative” effect, while the remainder (*n* = 2) were “unsure” of its potential effects.

Only one of the nine participants (11%) correctly identified the session on which they received CBD; three (30%) correctly identified the session on which they received the placebo. When participants were correct, they were only “somewhat” confident in their prediction. Participants were either incorrect (*n* = 6) or unsure (*n* = 8) which treatment they received during the remaining trials.

### Adverse Events

No serious adverse events occurred during this trial. One participant fainted during the Baseline (pre-treatment) blood draw; this treatment session was terminated and rescheduled.

## Discussion

This pilot study investigated the effects of acute, oral CBD treatment (300 mg) *versus* placebo on physiological and psychological responses to aerobic exercise in a small sample of male endurance-trained runners. Effects were considered worthy of further investigation if the 85% CI around Cohen’s d_z_ included ± 0.5 but not zero. Results suggest that CBD may alter several key parameters, including submaximal (V̇O_2_) and maximal (V̇O_2max_) oxygen consumption, feelings of pleasure during submaximal exercise and markers of exercise-induced inflammation. However, it is important to stress that the analytical approach employed is susceptible to Type I error (i.e. identifying “false” effects) and was selected to inform the design of future “definitive” trials [23]. These initial observations should, therefore, be interpreted with caution and not taken as compelling evidence of “effect”.

CBD appeared to increase V̇O_2_ (and V̇CO_2_) during 60 min of submaximal exercise (RUN 1). An increase in V̇O_2_ (at a fixed workload) may suggest a reduction in running economy, and therefore, a detrimental effect of CBD on aerobic exercise performance. However, the difference was relatively subtle (37_EX_: + 38 ± 48 mL·min^−1^, 50_EX_: + 26 ± 55 mL·min^−1^) compared to that of other interventions known to influence V̇O_2_ (e.g. ketogenic diets: + 90—140 mL·min^−1^ [40]; dietary nitrates: − 143 mL·min^−1^ [41]), corresponding to only a 2.3% increase in estimated energy expenditure (+ 82 ± 121 kJ; based on the equations of Frayn [42]). Furthermore, it was not accompanied by an increase in HR or RPE, nor a decrease in TTE as one might anticipate [40]. In fact, in addition to V̇O_2_, CBD appeared to increase V̇O_2max_ (+ 119 ± 206 mL·min^−1^; + 1.5 ± 2.8 mL·min^−1^·kg^−1^). Thus, if present, any detrimental effect of CBD on running economy appears to be small and seems unlikely to impair aerobic exercise performance.

The potential mechanism by which CBD might have affected V̇O_2_ responses in the present study is difficult to predict given its multiple pharmacological actions [22] and the limited amount of research in this field. Nonetheless, some mechanisms can be excluded. First, the current findings (and some previous research [43]) suggest that alterations in breathing patterns (i.e. RR, *V*_T_, *V*_E_) are unlikely to explain the observed increase in V̇O_2_. Second, while participants could have modified their running biomechanics on the treadmill (e.g. stride frequency, strike pattern, ground contact time), changing muscle recruitment and therefore V̇O_2_, this would not be expected to increase V̇O_2max_. Third, while preferential utilisation of fat as a metabolic substrate can increase V̇O_2_ [40], CBD did not appear to decrease submaximal RER. Fourth, while impairments in mitochondrial function (e.g. increased non-energetic proton leak across the inner membrane) can increase V̇O_2_ [44], this would not be expected to increase V̇O_2max_ [45] (and would likely decrease TTE). Furthermore, most in vivo studies find that CBD improves aspects of mitochondrial function [46–49], although some in vitro (cellular) studies, usually involving high concentrations of CBD (e.g. > 5 µM), have reported detrimental effects [50–54].

One possible explanation for the observed increase in V̇O_2_ and V̇O_2max_ is that CBD increased tissue blood flow via vasodilation. Indeed, studies have shown that nitrate- and ascorbic acid-induced vasodilation can increase skeletal muscle blood flow and V̇O_2_ during hand-grip exercise [55, 56]. Findings from several preclinical studies also suggest that in vitro CBD treatment (i.e. ≤ 2-h exposure to 1–10 µM) can induce vasodilation in isolated arteries of rats [57–59] and humans [60]. A small clinical trial (*n* = 9) further reported that CBD (600 mg) reduced resting systolic BP (~ 6 mmHg) and total peripheral resistance during hand-grip exercise in normotensive males [61]. This mechanism could also explain why the observed increase in V̇O_2_ was not accompanied by a clear increase in HR or RPE; nor decrease in TTE, as described above. That said, BP did not appear to be affected in the current trial.

In terms of psychological effects, CBD appeared to improve affect (i.e. ratings of pleasure) during the first 40-min of submaximal exercise (20_EX_: + 0.7 ± 0.9; 40_EX_: + 0.8 ± 1.1) but with no difference at 60_EX_. It is important to note that the ratings obtained at 60_EX_ may have been influenced by the expectation of completing the exercise bout [62]; that is, participants “felt better” at 60_EX_ because they knew they were close to finishing the exercise task. Indeed, affect, which often decreases as exercise progresses [62], tended to increase from 40 to 60_EX_ on both treatments (Placebo: + 0.8 ± 1.5; CBD: + 0.1 ± 0.3), particularly placebo. Thus, the results at 60_EX_ should be interpreted with caution. The absence of any anxiolytic or other mood-altering effect of CBD should also be interpreted with caution as all participants recorded very low absolute scores on the STAI-S and POMS.

The mechanism via which CBD may improve affect during exercise is also difficult to predict. CBD has been shown to interact with a complex network of receptors and signalling pathways involved in mood regulation (e.g. 5-HT_1A_, TRPV_1_, PPARy, the cannabinoid type 1 receptor [CB1] [22]). An alternative possibility is that CBD, which has demonstrated some analgesic potential in humans [63], might have attenuated subjective feelings of pain, thereby increasing feelings of pleasure during exercise in the present study.

Exercise increased the serum concentrations of various biomarkers of systemic inflammation (i.e. TNF-α, IL-1β, IL-6), muscle damage (i.e. Mb, CK) and gastrointestinal damage (i.e. LPS) in the current trial. CBD appeared to suppress the exercise-induced increase in pro-inflammatory cytokine, IL-1β, Post-RUN 2 and 1-h Post-RUN 2. This is consistent with the robust anti-inflammatory effects of CBD in animal models [64]. However, it should be noted that (even post-exercise) serum IL-1β concentrations were very low (often needing to be extrapolated from the standard curve); effects should therefore be interpreted with caution. In addition, neither IL-6 nor TNF-α concentrations appeared to be impacted by CBD. Regarding muscle damage, CBD appeared to increase Mb concentrations 1-h Post-RUN-2, suggesting an exacerbation of muscle damage. This unexpected change could have been driven by a small increase in TTE (+ 39 ± 85 s) observed on the CBD treatment. While there is preliminary evidence suggesting that CBD may protect against some forms of GI damage [65], it did not appear to influence LPS concentrations in the current trial. Further research, employing more demanding exercise protocols (e.g. heat stress, eccentrically loaded exercise), may be required to better understand the anti-inflammatory and protective effects of CBD.

Several aspects of the current trial appeared to influence plasma AEA concentrations. First, all nine participants’ plasma AEA concentrations: (1) decreased from Baseline to Pre-RUN 1; and (2) increased from Pre-RUN 1 to Post-RUN 1, regardless of the treatment administered. These effects are likely to be due to breakfast consumption and the completion of submaximal exercise (RUN 1), respectively. Indeed, previous studies report that circulating AEA concentrations decrease post-prandially [66] and increase following submaximal exercise (e.g. ~ 70–85% HR_max_) [67, 68] with the endocannabinoid system (in general) believed to contribute to the regulation of energy intake and storage [69]. Second, CBD appeared to decrease plasma AEA concentrations Post-RUN 2 relative to placebo. The only other study to have investigated the effect of CBD on circulating endocannabinoids, in fact, observed the *opposite* effect; that is, chronic CBD treatment (800 mg∙d^−1^; 4 weeks) increased (resting) serum AEA concentrations in patients with schizophrenia [8]. Of course, the participant population, dosing regimen and experimental paradigm differed greatly between studies. While it is difficult to predict the mechanism by which CBD might have influenced AEA (and we cannot “rule out” an effect of the small increase in TTE observed on the CBD treatment), these findings add to a small body of evidence suggesting that CBD may modulate endocannabinoid tone.

This investigation does contain several limitations. First, as indicated above, the pilot trial was not formally powered to assess “effect”. It also generated a number of *uncertain* results; however, it is important to recognise that some degree of “uncertainty” is also often present in *p*-values > 0.05. Second, only male participants were recruited in this initial pilot study. As various physiological processes are influenced by the menstrual cycle, including substrate metabolism [70], this decision was made to minimise normal session-to-session variability and therefore maximise our capacity to detect effect (if present), given the limited sample size. Future studies should investigate the impact of CBD on both male and female physiology. Third, some participants had detectable albeit low levels of CBD (*n* = 2; < 0.8 ng·mL^−1^) and(or) 7-COOH-CBD (*n* = 5; < 19.1 ng·mL^−1^) in plasma on their placebo trial after receiving the active treatment ≥ 7-days prior. Although these low concentrations are unlikely to have had a meaningful effect on our results, future studies should consider extending the washout period between sessions (bearing in mind that this may require further standardisation of training and exercise behaviour). Fourth, plasma CBD concentrations remained relatively low during the initial stages of submaximal exercise (RUN 1); future studies, using similar formulations, may therefore benefit from delaying the start of exercise to better capture the observed *T*_max_. Fifth, the CBD dose used in this investigation (300 mg) was relatively high (for a healthy population) and was selected to gain initial insights into CBD’s effects. Future investigations may consider using doses that more closely resemble products available to (and used by) the general and athlete population.

## Conclusion

These preliminary results suggest that acute, oral CBD treatment has the potential to alter key physiological and psychological responses during aerobic exercise. Indeed, its effects on V̇O_2_ responses, feelings of pleasure during exercise and exercise-induced inflammation appear worthy of further investigation. The absence of a clear detrimental effect on RPE, TTE and V̇O_2max_ also suggests that CBD is unlikely to impair aerobic exercise performance in endurance-trained males and may therefore have utility within the sporting context. Further research, involving a larger participant sample and different dosing regimens (e.g. chronic treatment, lower doses), is required to confirm and better understand these initial observations.

## Supplementary Information

**Additional file 1.** Methods and results.

## Data Availability

The datasets generated during and/or analysed during the current study are available from the corresponding author on reasonable request.

## Abbreviations

BG: Blood glucose
BL: Blood lactate
BP: Blood pressure
BW: Body weight
CBD: Cannabidiol
CHO: Carbohydrate
CI: Confidence interval
CK: Creatine kinase
EX: Minutes of exercise
GI: Gastrointestinal
HR: Heart rate
HR_max_: Maximal heart rate
IL-1β: Interleukin-1β
IL-6: Interleukin-6
LPS: Liposaccharide
Mb: Myoglobin
MCT: Medium chain triglyceride
POMS: Profile of mood states
RER: Respiratory exchange ratio
RER_max_: Maximal respiratory exchange ratio
RR: Respiratory rate
SD: Standard deviation
SE: Standard error
STAI-S: Short Form State-Trait Anxiety Inventory
THC: Tetrahydrocannabinol
TNF-α: Tumour necrosis factor-α
TTE: Time to exhaustion
U_SG_: Urine-specific gravity
V̇CO_2_: Volume of carbon dioxide
V̇CO_2max_: Maximal volume of carbon dioxide
*V*_E_: Minute ventilation
V̇O_2_: Volume of oxygen
V̇O_2max_: Maximal volume of oxygen
*V*_t_: Tidal volume
