# Supplementary material for: Effects of Cannabidiol on Exercise Physiology and Bioenergetics: A Randomised Controlled Pilot Trial
Source: Sports Med Open. 2022 Mar 2;8:27. doi: 10.1186/s40798-022-00417-y (PMC8891421; doi:10.1186/s40798-022-00417-y)
Supplement: Supplementary file 1 — Additional file 1. Methods and results. [file 40798_2022_417_MOESM1_ESM.docx]

**Effects of Cannabidiol on Exercise Physiology and Bioenergetics: A Randomised Controlled Pilot Trial**

*Sports Medicine – Open*

Ayshe Sahinovic*^1,2,3^, Christopher Irwin^4^, Peter T. Doohan^1,2,3^, Richard C. Kevin^1,2,3^, Amanda J. Cox^5^, Namson S. Lau^6,7,8^, Ben Desbrow^4^, Nathan A. Johnson^8^, Angelo Sabag^9^, Matthew Hislop^10^, Paul S. Haber^8,11^, Iain S. McGregor^1,2,3^ and Danielle McCartney^1,2,3^

^1^Lambert Initiative for Cannabinoid Therapeutics, The University of Sydney, Sydney, New South Wales, Australia

^2^Brain and Mind Centre, The University of Sydney, Sydney, New South Wales, Australia

^3^School of Psychology, Faculty of Science, The University of Sydney, Sydney, New South Wales, Australia

^4^School of Health Sciences and Social Work, Griffith University, Gold Coast, Australia

^5^School of Medical Science, Griffith University, Gold Coast, Australia

^6^South West Clinical School, University of New South Wales, Sydney, Australia

^7^Ingham Institute of Applied Medical Research, Liverpool, Australia

^8^Faculty of Medicine and Health, University of Sydney, Sydney, Australia

^9^NICM Health Research Institute, Western Sydney University, Sydney, Australia

^10^Brisbane Sport and Exercise Medicine Specialists, Brisbane, Australia

^11^Royal Prince Alfred Hospital, Sydney, Australia

**Corresponding author:**

Ayshe Sahinovic

Lambert Initiative for Cannabinoid Therapeutics

Brain and Mind Centre, University of Sydney

Camperdown NSW, Australia 2050.

Email: [ayshe.sahinovic@sydney.edu.au](mailto:ayshe.sahinovic@sydney.edu.au)

**SUPPLEMENTARY MATERIAL #1:** Participant Eligibility Criteria

**Inclusion Criteria**

1. Males ≥18 and ≤45 years of age;
2. Endurance-trained individuals; i.e., who have run an average of ≥40 km·wk^-1^ for the last month (or more) and can sustain moderate intensity running exercise for >60-minutes;
3. No reported use of cannabis or cannabinoids within the past 3 months; to be confirmed by a negative urine drug screen (UDS) at the medical screening; and
4. Proficient in English (i.e., must not require an English translator).

**Exclusion Criteria**

1. Cannabis dependence or any other drug or alcohol dependence, as per the International Statistical Classification of Diseases 10th Revision (ICD)-10 criteria or at a medical doctor’s discretion;
2. A history (self-reported) of allergic reaction (e.g., rhinitis, urticaria, contact dermatitis, anaphylaxis) to cannabis, cannabis products or cannabinoids;
3. A history (self-reported) of a clinically significant adverse response to cannabidiol (CBD);
4. A history of a major psychiatric disorder within the previous 12 months, as per the Diagnostic and Statistical Manual of Mental Disorders (DSM)-V criteria or at the medical doctor’s discretion, except, mild to moderate depression (score <20 on the Beck Depression Inventory [BDI] or mild to moderate anxiety (score <16 on the Beck Anxiety Inventory [BAI]
5. A history of attempted suicide or current suicide ideation as determined by a score >0 on Question 9 of the Patient Health Questionnaire (PHQ)-9
6. A (self-reported) history of, or current, cardiovascular, respiratory, renal, neurological, gastrointestinal, or endocrinological disorders;
7. A major illness or injury that interrupted their usual training routine for a period of ≥3 weeks during the past 3 months;
8. Inability to refrain from consuming alcohol (24 h) and caffeine (12 h) prior to each experimental trial;
9. Inability to refrain from using anti-inflammatory medications (24 h) prior to each experimental trial;
10. Inability to refrain from using other central nervous system active drugs (e.g., cannabis, opioids, benzodiazepines) while participating in this project;
11. Use of medications that may influence CBD metabolism (e.g., inducers or inhibitors of the CYP450 enzyme system);
12. Use of medications handled by transporter proteins or CYP enzymes that are inhibited by CBD, such as anticoagulants, calcium channel blockers, beta blockers, sulfonylureas and anti-convulsant; and
13. Required to complete mandatory drug testing for cannabis (e.g., workplace testing, antidoping in sport testing).

**SUPPLEMENTARY MATERIAL #2:** STAI-S and POMS Measurements

**State-Trait Anxiety Inventory (STAI-S) Measurements**

During the STAI-S, participants indicated how ‘Calm’, ‘Tense’, ‘Upset’, ‘Relaxed’, ‘Content’ and ‘Worried’ they felt on a 4-point Likert scale, where 1 represented “not at all” and 4, “very much”. After reversing the scores on positive items, the total score was summed and multiplied by ‘(20 $\div$ 6)’ to generate a result comparable to that obtained on the 20-item STAI [1]. Higher STAI-S scores represent a more negative emotional state.

**Profile of Mood States (POMS) Measurements**

The POMS consisted of 40-items loaded on seven subscales: ‘Tension’, ‘Depression’, ‘Fatigue’, ‘Confusion’, ‘Anger’, ‘Vigour’ and ‘Esteem’. Participants rated their experience of each item on a 5-point Likert scale, where 0 represented “not at all” and 4 represented “extremely”. Scores were calculated as described elsewhere [2]. Total mood disturbance (TMD) was calculated by subtracting the sum of positive emotional states (Vigour and Esteem) from negative states (Tension, Depression, Fatigue, Confusion and Anger) and adding 100 [2]. Thus, higher TMD scores represent a more negative emotional state (i.e., greater mood disturbance).

1. Marteau TM, and Bekker H. The development of a six-item short-form of the state scale of the Spielberger State-Trait Anxiety Inventory (STAI). *Br J Clin Psychol* 31: 301-306, 1992.

2. Grove, J.R. and H. Prapavessis, *Preliminary Evidence for the Reliability and Validity of an Abbreviated Profile of Mood States.* International Journal of Sport Psychology, 1992. **23**(2): p. 93-109.

**SUPPLEMENTARY MATERIAL #3:** Plasma Anandamide Analysis

**Sample Preparation for Anandamide (AEA) Analysis**

Frozen plasma samples were thawed at room temperature and placed on ice as they finished thawing. Aliquots of 200μL were transferred to 1.5 mL capacity polypropylene centrifuge tubes and spiked with 5μL of an internal standard solution (containing arachidonoyl ethanolamide-d4 (250 ng/mL). Similar to validated methods described by Gurke et al.(1), AEA was isolated via a two-phase liquid-liquid extraction where 400μL ethylacetate:hexane (9:1) was added to each spiked plasma sample. Samples were then vortexed for 20 mins and centrifuged at 18,000×g for 20 mins at 4°C. The resulting organic supernatant was transferred to a new glass culture tube and dried to completeness under nitrogen gas. Samples were reconstituted in 17.5μL of 0.1% formic acid and 32.5μL of methanol for immediate analysis (see below). Calibrator and quality control samples were prepared in 200μL phosphate buffered saline spiked to known analyte concentrations and prepared in tandem with experimental samples as described above. Samples remained on ice throughout the preparation.

**Sample Analysis**

AEA was analysed by ultra-high performance liquid chromatography/ mass spectrometry (UHPLC-MS/MS) using a Shimadzu LC-30AD UHPLC system (Kyoto, Japan) coupled to a Shimadzu 8040 triple quadrupole mass spectrometer. Samples were separated on a Waters ACQUITY UPLC CSH C18 analytical column (100 × 2.1 mm, 1.7μm particle size) (Milford, MA, USA) equipped with a Waters ACQUITY UPLC BEH C18 guard column (5 × 2.1 mm, 1.7μm particle size), using gradient elution and an injection volume of 20μL. Mobile phases 0.1% formic acid in water (A) and methanol (B) were delivered at a flow rate of 0.3 L/min as follows: mobile phase B was first held at 65% for 0.5 mins, then increased from 65% to 82% over 0.1 mins, held at 82% for 11.4 mins, then increased from 82% to 100% over 0.1 mins, held at 100% for 1 min then decreased back to 65% over 0.1 mins and equilibrated at 65% for 2 mins. The MS device was operated in multiple reaction monitoring (MRM) mode using electrospray ionization in positive mode (ESI+), with a capillary voltage of 4.5 kV, a source temperature of 450°C, and a desolvation temperature of 100°C and a column temperature of 40°C. Nebulising gas and drying gas flow rates were 3 L/min and 15L/min, respectively. Mass transitions and collision energies were optimised for each analyte and internal standard individually, using dwell times of 50 or 100ms (Table S1). Chromatographic separation was also optimised for the analyte set and lower limit of quantification (LLOQ) values were determined (Table S1).

**Table S1.** Mass spectrometric parameters for AEA analysis

LLOQ: lower limit of quantification, *: Quantifier transition

| **Analyte** | **Abbreviation** | **Molecular Weight (mol·g^-1^)** | **Mass Transitions**  **(m/z)** | | | **Collision Energy (eV)** | **Retention time**  **(mins)** | **LLOQ (ng/mL)** |
| --- | --- | --- | --- | --- | --- | --- | --- | --- |
| Arachidonoyl ethanolamine | *AEA* | 347.5 | 348.35 | → | 62.15* | -14 | 6.55 | 0.05 |
|  |  |  | 348.35 | → | 91.15 | -47 |  |  |
| Arachidonoyl ethanolamide-d_4_ | *d_4_-AEA* | 351.6 | 352.35 | → | 66.25* | -14 | 6.53 | - |
|  |  |  | 352.35 | → | 91.15 | -47 |  |  |
|  |  |  | 313.30 | → | 70.20 | -35 |  |  |

**Reference**

1. Gurke R, Thomas D, Schreiber Y, Schäfer SMG, Fleck SC, Geisslinger G, and Ferreirós N. Determination of endocannabinoids and endocannabinoid-like substances in human K3EDTA plasma - LC-MS/MS method validation and pre-analytical characteristics. *Talanta* 204: 386-394, 2019.

**SUPPLEMENTARY MATERIAL #4:** Serum Biomarker Analysis

Stored serum samples were analysed to determine circulating interleukin (IL)-1β (HSLB00D; RnD Systems, Minneapolis, USA), IL-6 (HS600C; RnD Systems, Minneapolis, USA), Tumour necrosis factor α (TNFα; DY210-05; RnD Systems, Minneapolis, USA), Myoglobin (ab108652, Abcam, Cambridge, UK), Creatine Kinase (ab264617, Abcam, Cambridge, UK) and Claudin-3 (NBP2-75328; Novus Biologicals, Centennial, USA) concentrations using commercially available enzyme-linked immunosorbent assay (ELISA) kits. Circulating lipopolysaccharide (LPS) was determined using the limulus amebocyte lysate (LAL) chromogenic endpoint assay (HIT302; Hycult Biotech, Uden, The Netherlands). All assays were completed in accordance with the manufacturers’ instructions. All samples were analysed in duplicate, with the exception of IL-1β where low detected concentrations required samples to be re-analysed using the remaining assay plate; replicates were not possible to ensure all samples could be included for analysis. For each assay, all samples from a single participant were included on one assay plate to negate any inter-assay effects. The mean intra-assay coefficient of variation for sample replicates was for: IL-6 6.2%; TNFα 4.3%; Myoglobin 6.1%; CK 6.7%; Claudin-3 9.5%; and LPS 3.6%. Inter-assay coefficient of variation was for: IL-6 7.0%; TNFα 10.1%; Myoglobin 6.5%; CK 1.5%; Claudin-3 2.0%; and LPS 6.3%.

**SUPPLEMENTARY MATERIAL #5:** Serum Biomarker Concentrations

**Figure S1**. Serum biomarkers measured at Baseline, Pre- and Post-RUN 1, Post-RUN 2, and 1-hour Post-RUN 2. **A.** Tumour Necrosis Factor- α (TNF- α) (expressed as % change relative to baseline); **B.** Interleukin-1ß (IL-1ß); **C.** Interleukin-6 (IL-6); **D.** Myoglobin; **E.** Creatine Kinase (CK); **F.** Liposaccharide (LPS); **G.** Claudin 3. Analyses were conducted using a 2 (Treatment) x 3 (Time) repeated-measures ANOVA and post hoc comparisons were performed with paired *t*-tests. Values are mean ± SEM for placebo (white squares) and CBD (green circles) (*n* = 9). a, Differs from Baseline (*p*<0.05); b, Differs from RUN 1 (p<0.05).

**SUPPLEMENTARY MATERIAL #6:** Gastrointestinal (GI) Comfort Visual Analogue Scales (VAS) Ratings

**Figure S2:** Gastrointestinal comfort as measured using 100 mm visual analog scales (VASs) at Baseline, Pre- and Post-RUN 1, Post-RUN 2, and 1-hour Post-RUN 2. **A.** Abdominal Pain; **B.** Nausea; **C.** Heartburn; **D.** Regurgitation; **E.** Belching; **F.** Bloating and **G.** Flatulence. 0 mm represents “not at all” and 100 mm, “extremely”. Values are Median ± Interquartile Range for placebo (white squares) and CBD (green circles) (*n* = 9).

**SUPPLEMENTARY MATERIAL #7a:** State Trait Anxiety Inventory (STAI) score and Profile of Mood States (POMS) Total Mood Disturbance (TMD)

**Figure S3: A**. State Trait Anxiety Inventory (STAI-S) score and **B.** Profile of Mood States (POMS) Total Mood Disturbance (TMD) as measured at Baseline, Pre- and Post-RUN 1, Post-RUN 2, and 1-hour Post-RUN 2. The STAI-S score was calculated by taking participants ratings of how ‘Calm’, ‘Tense’, ‘Upset’, ‘Relaxed’, ‘Content’ and ‘Worried’ they felt on a 4-point Likert scale, where 1 represented “not at all” and 4, “very much”. After reversing the scores on positive items, the total score was summed and multiplied by ‘(20 $\div$ 6)’ to generate a result comparable to that obtained on the 20-item STAI. Higher STAI-S scores represent a more negative emotional state. Total mood disturbance (TMD) was calculated by

subtracting the sum of positive emotional states rated on the POMS subscales (vigour and esteem) from negative states (tension, depression, fatigue, confusion and anger) and adding 100. Higher TMD scores represent a more negative emotional state (i.e., greater mood disturbance).Values are mean ± SEM for placebo (white squares) and CBD (green circles) (*n* = 9).

**SUPPLEMENTARY MATERIAL #7b:** Profile of Mood States (POMS) ratings

**Figure S4.** Profile of Mood States (POMS) subscale ratings as measured at Baseline, Pre- and Post-RUN 1, Post-RUN 2, and 1-hour Post-RUN 2. The POMS consisted of 40-items which were rated on a 5-point Likert scale where 0 represented ‘not at all’ and 5 represented ‘extremely’ and grouped into the following subscales: **A.** Tension ratings; **B.** Depression ratings; **C.** Anger ratings; **D.** Vigour ratings; **E.** Fatigue ratings; **F.** Confusion ratings; **G.** Esteem ratings. Values are mean ± SEM for placebo (white squares) and CBD (green circles) (*n* = 9).

**SUPPLEMENTARY MATERIAL #8:** Seated Systolic and Diastolic Blood Pressure

**Figure S5.** **A.** Systolic and **B.** diastolic blood pressure (BP) measured at Baseline, Pre- RUN 1, Post-RUN 1 and Post-RUN 2. Values are mean ± SEM for placebo (white squares) and CBD (green circles) (*n* = 9).
